# Goblet Cells and Mucus Composition in Jejunum and Ileum Containing Peyer’s Patches and in Colon: A Study in Pigs

**DOI:** 10.3390/ani15192852

**Published:** 2025-09-29

**Authors:** Vladimir Ginoski, José Luis Cortés Sánchez, Stefan Kahlert, Johannes Schulze Holthausen, Łukasz Grześkowiak, Jürgen Zentek, Hermann-Josef Rothkötter

**Affiliations:** 1 Institute of Anatomy, Faculty of Medicine, Otto von Guericke University Magdeburg, Leipziger Street 44, Building 43, 39120 Magdeburg, Germany; vladimir.ginoski@med.ovgu.de (V.G.); jose.cortes@med.ovgu.de (J.L.C.S.); stefan.kahlert@med.ovgu.de (S.K.); 2 Institute of Animal Nutrition, Department of Veterinary Medicine, Freie Universität Berlin, 14195 Berlin, Germany; johannes.holthausen@fu-berlin.de (J.S.H.); lukasz.grzeskowiak@fu-berlin.de (Ł.G.); juergen.zentek@fu-berlin.de (J.Z.)

**Keywords:** mucins, goblet cells, Peyer’s patches, jejunum, ileum, colon, follicle-associated epithelium, pigs

## Abstract

The intestinal mucus layer is a dynamic protective barrier that maintains gut homeostasis, supports immune defense, and regulates host–microbiota interactions. Rodent models have yielded valuable insights, but their intestinal structure and physiology differ from those of humans and pigs. By contrast, the omnivorous pig shares closer anatomical and immunological features with humans, making it a relevant large-animal model in translational studies. In this study, we established a histological workflow for porcine intestine by combining Carnoy’s fixation with Alcian Blue–Periodic Acid–Schiff and Mucicarmine staining. This enabled accurate visualization and quantification of goblet-cell density and mucus thickness across intestinal segments, with a particular focus on Peyer’s patches—key sites of immune surveillance. Both stains produced consistent results. We observed a clear proximal-to-distal gradient, from jejunum to colon, in mucus thickness: the colon displayed the thickest layer (~100 μm), whereas the follicle-associated epithelium over Peyer’s patches in the jejunum and ileum showed a markedly thinner layer (<12 μm) and fewer goblet cells. Immunofluorescence further revealed strong cytokeratin-18 expression in goblet cells, delineating their morphology and polarity. These findings demonstrate region-specific differences in mucus architecture and goblet-cell distribution that likely reflect specialized immune functions, advancing our understanding of the intestinal barrier and informing future strategies to support gut health and immunity.

## Simple Summary

A mucus layer lines the gut, serving as a barrier against harmful bacteria while providing a habitat for beneficial microbes. This barrier is produced by goblet cells, specialized cells that also support mucosal immune defense. In this study, we examined weaned pigs, whose gastrointestinal systems closely resemble those of humans, with particular attention to Peyer’s patches—intestinal “checkpoints” for immune surveillance. Using two histochemical staining methods, we quantified mucus thickness and goblet-cell density across different gut segments. Mucus was thinnest over Peyer’s patches and much thicker in the large intestine; likewise, goblet-cell counts were lower in Peyer’s-patch regions than in adjacent tissue. The two methods yielded closely similar results, supporting their reliability. These findings reveal regional variation in the mucus barrier and identify sites where the immune system may have closer contact with intestinal contents. Understanding these patterns could inform dietary and medical strategies to support digestive health in both humans and pigs.

## 1. Introduction

The intestine must balance two essential functions: nutrient absorption and immune defense. Gastrointestinal barrier function is maintained through coordinated regulation of the mucus layer, epithelial cell activity, and the mucosal immune system [1]. This surface protection is critical because the human gastrointestinal tract (GIT) harbors dense microbial communities—up to ~10^13^–10^14^ bacteria per gram of intestinal content (gIC) in the colon. Bacterial numbers increase along the tract, from ~10^2^/gIC in the stomach to ~10^8^/gIC in the distal ileum and ~10^11^–10^14^/gIC in the large intestine [1,2,3]. An human individual’s gut microbiome typically contains approximately 200–1000 microbial species [3]. In weaning piglets, the lower intestine (cecum–rectum) contains ~10^10^ bacterial 16S rRNA gene copies per gram, whereas the upper gut contains ~10^7^–10^9^/gene copies per gram [4]. Gut microorganisms shape immune system development, synthesize vitamins, and help break down complex dietary carbohydrates, contributing up to ~10.7–24.2% of the host’s energy intake [5,6]. Spatial differences in bacterial load imply that the mucin gel layer may vary in thickness and possibly composition across intestinal segments. The mucus constitutes a complex, dynamic interface that mediates host–microbe interactions [7].

Mucins are large, heavily glycosylated proteins secreted by intestinal goblet cells (GCs) that form the protective mucus barrier over the epithelium. Their polymeric backbones are densely loaded with carbohydrate-rich *O*-linked glycans, which account for ~80–90% of mucin mass. This structure protects the epithelial surface from possible dehydration and mechanical stress and guards against luminal microbes, toxins, and antigens [7]. Their carbohydrate moieties include neutral sugars (e.g., galactose, fucose), hexosamines (e.g., *N*-acetylgalactosamine), and sialic acids, which together confer structural and functional diversity to mucins [7]. Histochemically, GCs are classified by mucin composition into neutral (uncharged), acidic (negatively charged), and mixed phenotypes that produce both [8,9]. Acidic mucins are subclassified into sialomucins and sulfomucins, depending on whether sialic acid or sulfate groups predominate. Compared with neutral mucins, acidic mucins are generally more resistant to degradation and enzymatic hydrolysis by host enzymes and commensal bacteria, underscoring their role in maintaining mucosal integrity [5,10]. Mucins occur as membrane-bound glycoproteins that contribute to the epithelial glycocalyx (e.g., MUC1, MUC4) and as secreted mucins, which include gel-forming (e.g., MUC2) and non-gel-forming (e.g., MUC7) types [1,11,12,13]. The principal gel-forming mucin in intestinal mucus is MUC2, a large (~5200–amino-acid) glycoprotein whose multimeric, net-like architecture creates an insoluble gel [14]. Other bioactive constituents of intestinal mucus include secretory IgA; trefoil factors; antimicrobial peptides (e.g., lysozyme, defensins, cathelicidins); phospholipase A_2_; and α1-antitrypsin [1,15]. Studies in mice and humans reveal a distinct bilaminar organization of intestinal mucus, comprising inner and outer layers [14]. The stomach and colon feature a double-layered mucus hydrogel: a dense, insoluble inner layer that adheres tightly to the epithelium, and a looser, bacteria-rich outer layer generated by proteolytic modification and expansion of the inner layer [7,16]. The gut microbiota promotes conversion of the inner mucus layer into the outer layer. In the small intestine, the mucus is comparatively thin and irregular, accumulating mainly at the tips of villi [1,3,14,17,18,19,20,21].

The intestinal epithelium is a simple columnar, highly specialized, and polarized monolayer that forms a continuous barrier between the gut lumen and the immune-cell-rich lamina propria. This dynamic layer comprises absorptive enterocytes (colonocytes in the colon), GCs, Paneth cells, tuft cells, cup cells, enteroendocrine cells, and microfold (M) cells—each continuously renewed from crypt-base stem cells via lineage-specific gene programs [2,15,22,23]. The intestinal epithelium is protected by goblet cell-derived mucus and secretory IgA (sIgA), which is transcytosed via the polymeric immunoglobulin receptor (pIgR) and incorporated into the mucus layer. Together with other epithelial effectors—such as Paneth cell-derived α-defensins and lysozyme—IgA and mucus provide both nonspecific and antigen-specific protection against luminal antigens [15].

The *lamina propria*—located beneath the epithelial basement membrane—constitutes a third line of gut defense. It maintains intestinal homeostasis by balancing immune tolerance with protective responses, largely through gut-associated lymphoid tissue (GALT) [24,25]. GALT is the body’s largest immune organ, housing approximately 60–70% of lymphocytes and serving as a central hub of mucosal immune defense [24,25,26]. GALT comprises Peyer’s patches (PPs), mesenteric lymph nodes, the appendix, isolated lymphoid follicles, and diffuse GALT, the latter consisting of dispersed T and B cells, macrophages, eosinophils, basophils, and mast cells throughout the lamina propria [24,27,28]. Peyer’s patches (PPs) are lymphoid aggregates located along the antimesenteric border of the porcine small intestine (jejunum and ileum), increasing in number from proximal to distal segments. Structurally, they comprise three regions: the follicular area, the interfollicular region, and the follicle-associated epithelium (FAE). The FAE is a specialized epithelium overlying the lymphoid tissue; it contains enterocytes, M cells specialized for antigen uptake, intraepithelial lymphocytes, dendritic-cell processes, and B and T cells [27,28]. M cells translocate luminal antigens to the underlying immune tissue of the dome area (DA), though their role in transporting larger dietary proteins remains uncertain. In pigs, M cells express cytokeratin 18 (CK18), consistent with an epithelial lineage and a cytoskeletal architecture suited for rapid transcytosis [29].

Given the central role of PPs in inducing oral immunity and tolerance to dietary antigens, we hypothesized that goblet-cell density and mucus-layer properties differ within PPs-associated regions. We therefore compared goblet-cell distribution and mucus thickness between normal intestinal mucosa and the mucosa overlying PPs. The study was conducted in weaned pigs—an omnivorous large-animal model with close anatomical and immunological similarity to humans [30]. We tested how Carnoy’s fixation combined with Alcian Blue–Periodic Acid–Schiff (AB–PAS) and Mucicarmine (MC) staining enables accurate visualization of goblet cells and reliable quantification of mucus-layer thickness across porcine intestinal segments, applying both methods to compare performance, cross-validate results, and reduce bias.

## 2. Materials and Methods

### 2.1. Animals

The animal samples originated from three studies. The first was approved by the Regional Office for Health and Social Affairs Berlin (LAGeSo; StN 023/21) and conducted in accordance with EU Directive 2010/63/EU and Commission Recommendation 2007/526/EC. In this trial, 56 nine-week-old pigs (DanBred × Duroc; 21.8 ± 4.1 kg body weight [BW] at week 9, reaching a final BW of ~36–37 kg at week 12; 31 males and 25 females) were randomly assigned and fed an isocaloric, isonitrogenous diet for three weeks. The diet met or exceeded the recommendations of the Society of Nutrition Physiology. Full study details are provided in Saliu et al. [31]. Only these samples were included in the histochemical and statistical analyses.

The second study was also approved by the State Office of Health and Social Affairs Berlin (LAGeSo; T 0063/19) and conducted in accordance with German ethical guidelines. Eighty-eight German Landrace piglets of mixed sex were weaned at 28 days of age (8.3 ± 1.1 kg initial BW) and fed a diet meeting or exceeding nutritional recommendations. From the 88 animals, sacrificed at 8.7 weeks of age (~20–22 kg BW), we obtained a total of 92 jejunal tissue cryo-samples originating from 44 individuals, comprising 22 females and 22 males. Feed and water were provided ad libitum, and health status was monitored daily. Tissues were snap-frozen in liquid nitrogen and stored at −80 °C. Complete study details are available in Ellner et al. [32].

The third study was conducted at the Friedrich-Loeffler-Institute in Braunschweig, Germany, with approval from the Lower Saxony State Office for Consumer Protection and Food Safety (file no. 33.4-42502-04-13/1274), in accordance with the German Animal Welfare Act and EU regulations. Forty-one German Landrace barrows (castrated male pigs; weaned at 4.7 weeks, with an initial body weight [BW] of 25.8 ± 3.7 kg at week 5 of age) were euthanized on day 29 of the trial (Bannert et al. [33]). At sacrifice (~60 days, ~9 weeks of age), they had a mean BW of 40.5 ± 3.0 kg. From the 41 animals, we obtained a total of 51 colon cryo-samples from the control group [33].

Histochemistry was performed on samples from the first study only (*n = 6* animals for each intestinal segment analyzed). In three jejunal samples from the second study, we conducted indirect immunofluorescence (IF) to examine the microtopography of the jejunal wall. Three animal tissue samples from the third study were used to analyze cytokeratin-18 (CK18) expression in the colon.

### 2.2. Tissue Sampling for Histochemistry

Tissue for histochemistry was sampled from multiple regions of the GIT. Small-intestine segments (0.5–1 cm) were collected from the jejunum (~1 m distal to the *flexura duodenojejunalis*) and the ileum (~10 cm distal to the point where PPs become macroscopically continuous), as well as from the middle of the colon. Specimens were obtained without opening the intestinal lumen to preserve the native organization and thickness of the surface mucus gel layer. Tissues were briefly rinsed in ice-cold saline to remove gross luminal contents and then opened along the mesenteric border. Approximately 7 × 7 × 7 mm pieces were fixed in Carnoy’s fixative (CARNOY Fixierlösung, MORPHISTO GmbH, Offenbach am Main, Germany) for 24 h at room temperature (RT).

Porcine jejunal and colonic tissues for indirect immunofluorescence (IF) were obtained from the studies described above. Tissue samples (~3 × 3 × 3 mm) were fixed, snap-frozen in liquid nitrogen, and stored at −80 °C in polypropylene tubes.

### 2.3. Histochemical Technique

Carnoy’s solution, a water-free fixative, is an especially effective method that minimizes the dehydration artifacts typically seen with other fixatives [34,35,36]. The solution is composed of 60% ethanol, 30% chloroform, and 10% glacial acetic acid (C_2_H_5_OH: 600 mL/L; CHCl_3_: 300 mL/L; C_2_H_4_O_2_: 100 mL/L). Following fixation, samples were briefly rinsed in ethanol to remove residual Carnoy’s solution components and immediately processed for paraffin embedding. Tissue processing was performed using the Histokinette Automatic Tissue Processor (Leica TP1020, Leica Biosystems GmbH, Nussloch, Germany). Samples were dehydrated in graded ethanol series (70% ethanol twice for 5 min and 5 h, followed by 80%, 90%, 96%, and 100% ethanol over a total duration of 20 h), then incubated in xylene twice for 2 h, and embedded transversely in paraffin (Paraplast^®^, Carl Roth GmbH + Co. KG, Karlsruhe, Germany) in two 6 h cycles. Once embedded (Leica EG1150 C Cold Plate for Modular Tissue Embedding System, Leica Biosystems GmbH, Nussloch, Germany), the tissue blocks were cooled to −20 °C and sectioned transversely using a sliding microtome (HM 400 R, MICROM Laborgeräte GmbH, Jena, Germany) at a thickness of 5 μm. Sections were first collected in a room-temperature (~20–22 °C) and then transferred to a warm-water bath (~40–45 °C) to flatten and facilitate smooth mounting on glass slides (Epredia™ Microscope Slides, Kalamazoo, MI, USA). Mounted slides were dried for 2 h at approximately 37 °C prior to histological staining.

To distinguish between different mucin types based on their glycan composition and to assess the thickness of the mucus gel layer, two modified histochemical staining protocols were employed. The first was an Alcian Blue–Periodic Acid–Schiff (AB-PAS) staining protocol. This method combines Alcian Blue (AB) (1%, pH 2.5 in acetic acid, 60 min; Staining Kit, MORPHISTO GmbH, Offenbach am Main, Germany) for the detection of acidic mucins with Periodic Acid–Schiff reagent (PAS) (2 × 10 min; PAS-Reaction Kit, MORPHISTO GmbH) for staining neutral mucins. Back-staining in the AB-PAS protocol was performed using Mayer’s hematoxylin (MORPHISTO GmbH). The second protocol utilized Mucicarmine (MC) staining (Mucicarmine Stock Solution according to Mayer, 60 min; MORPHISTO GmbH), combined with a counterstain using Metanil Yellow for Herovici staining (3 min; MORPHISTO GmbH), enabling the visualization of all mucin types. Following staining, sections were dehydrated, cleared, and mounted with Entellan™ (Merck, Darmstadt, Germany), then cover-slipped (Menzel Deckgläser, 24 × 60 mm, Gerhard Menzel GmbH, Leverkusen, Germany). Staining was performed in two batches to minimize technical variation (The full modified staining protocol is provided in Supplementary Table S1 and Figure S1).

### 2.4. Analysis of the Histological Slides and Histomorphometry Measurements

**Figure 1:**
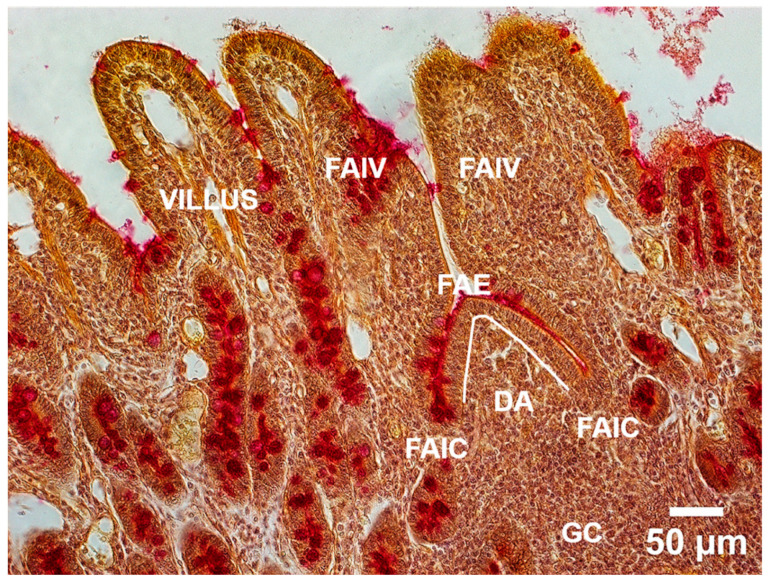
Mucicarmine (MC) Staining of Peyer’ patches (PPs) in porcine ileum with corresponding tissue segments measured in the study. (FAE—Follicle-associated epithelium marked with a white line, DA—“dome area” or subepithelial dome (SED), GC—germinative center, FAIC—Follicle associated crypt and FAIV—Follicle associated intestinal villus) (*Scale Bar* = 50 μm).

For each of the six animal samples, at least three slides were prepared and analyzed separately for each staining method, with each slide containing two tissue sections. Tissues stained with AB-PAS and MC were examined using a Zeiss Axioplan 2 imaging microscope (Carl Zeiss, Jena, Germany) equipped with a digital camera, Carl Zeiss AxioCam HRc (Carl Zeiss, Jena, Germany) and controlled by Zeiss Axiocam Software (AxioVs40, Version 4.8.2.0, 2006–2010, Carl Zeiss, Jena, Germany) for image analysis, at 200× magnification. Histomorphometric measurements were conducted on defined tissue segments, based on the depiction and description by Mantani et al. [37], and an example is shown in Figure 1:

- Follicle-associated epithelium (FAE) length was defined as the linear extent (in μm) of the epithelium overlaying the PPs in the dome area (DA), in both the ileum and jejunum. The boundaries of the FAE were demarcated by the junctional zones between the FAE and follicle-associated crypts (FAIC).
- Follicle-associated intestinal villus (FAIV) was identified as the first intestinal villus adjacent to the FAE within jejunal and ileal PPs. FAIV was delineated between the FAIV–FAIC and FAIV–ordinary crypt junctional zones and it was not included in the morphological analysis.
- Follicle-associated crypts (FAIC), located between the FAE and FAIV, were not included in the morphological analysis.
- Conventional jejunal and ileal villi were measured with the following parameters—height (tip to base) and width (at midpoint), excluding associated crypts. These villi were defined as mucosal projections located between two villus–crypt junctions. Crypt–villus junctions represent transitional zones into intestinal crypts. The measurements were performed using calibrated digital tools within the specified imaging software described below.
- In the colon, the depth (in μm) of three intact colonic crypts per sample was measured from the crypt opening at the basement membrane surface of the crypt base.

The open-source software Fiji (ImageJ2, v2.9.0; https://imagej.net/software/fiji/ accessed on 8 November 2024) was used for morphometric and cell quantification analysis. The scaling factor in Fiji ImageJ was calibrated using a stage micrometer to convert pixel values to known distances. Parameters measured included FAE length, height and width of regular villi, and colonic crypt depth. All parameters were measured in triplicate in six study animals; replicate values were averaged to obtain a single value per animal. Villus height and width were measured from three well-oriented, intact villi per slide. Colonic crypts were randomly selected from properly oriented tissue regions. Goblet cells were quantified and normalized per 100 μm of basement membrane. Goblet cell subtypes—neutral, acidic, and mixed mucins—were identified via histological image analysis based on staining characteristics: deep purple (mixed), blue (acidic), and magenta (neutral) in AB-PAS, and red to pink in MC staining. Absolute goblet cell counts were normalized per 100 μm of basement membrane in FAE, FAIV, regular villi (jejunum and ileum), and colonic crypts. The quantitative assessment of goblet cell counts was based on the rigorous criteria established by Baidoo et al. [38].

### 2.5. Mucus Thickness Measurement

Mucus thickness along the porcine GIT was assessed histologically. A representative region of the epithelium covered by a continuous mucus layer was selected, defined by a minimum of 100 μm in uninterrupted length and preservation of both epithelial and mucus layers. Mucus thickness was defined as the perpendicular distance from the outermost boundary of the mucus gel to the luminal surface of the epithelial lining [39]. Measurements were performed perpendicularly to the mucosal surface to ensure consistency. The mucin gel layer was measured perpendicularly in the regions of the PPs and colon (the intercrypt epithelium was taken as a reference point for the colon). In the jejunum and ileum, measurements were taken from the villus tips when intact, or alternatively from the lateral aspects of the villus tip, always oriented perpendicularly toward the intestinal lumen rather than the intervillous region. For each animal, three adjacent measurements were recorded per region: one at the point of maximum mucus thickness and two flanking measurements positioned 10 μm to the left and right. The average of these three values was used as the representative thickness for each sample. All measurements were expressed in micrometers (μm) (see Supplementary Figure S2).

### 2.6. Indirect Immunofluorescence (IF) for Cytokeratin 18 (CK18)

Porcine jejunal and colonic tissue samples (~ 3 × 3 × 3 mm) from six animals were snap-frozen in liquid nitrogen and cryosectioned at 5 μm thickness (Leica CM3050 S Cryostat, Leica Biosystems GmbH, Nussloch, Germany). Two sections were mounted per slide (Epredia™ SuperFrost Plus™ Adhesion slides, Kalamazoo, MI, USA), with additional slides for negative controls (incubated with secondary antibody only). Sections were air-dried for 2 h at RT, fixed in cold 1:1 acetone/methanol (−20 °C) (Carl Roth GmbH + Co. KG, Karlsruhe, Germany) for 5 min, and washed five times (7 min each) in 0.1% Tween-20 in TBS (Tris-buffered saline). For staining, slides were placed in a humid chamber (Custom Immunohistochemistry Microscope Slides Humid Chamber, Kartell^®^, Noviglio (MI), Italy), permeabilized for 5 min with 0.1% Triton™ X-100 (Product Number: T8787, Sigma-Aldrich Chemie GmbH, Taufkirchen, Germany) in 0.1% Tween-20/TBS, and outlined with a hydrophobic barrier pen (ImmEdge^®^ Hydrophobic Barrier PAP Pen (H-4000), Vector Laboratories, Inc., Newark, CA, USA). To block nonspecific binding, sections were incubated for 1 h at RT with 10% Normal Goat Serum (NGS, HISTOPRIME, Biozol, Hamburg, Germany, Normal Goat Serum, 1:100) in 0.1% Tween-20/TBS supplemented with 1% Bovine Serum Albumin (BSA, Bovine Serum Albumin A2058, Sigma-Aldrich Chemie GmbH, Taufkirchen, Germany). Primary antibody (Mouse Anti-Cytokeratin Peptide CK18 monoclonal IgG1 (C8541) CY–90; Sigma-Aldrich Chemie GmbH) was applied (1:10,000 in 0.1% Tween-20/TBS + 1% BSA) and incubated overnight at 4 °C in the dark without agitation. After five washes, a fluorophore-conjugated secondary antibody (Goat anti-Mouse IgG1, Alexa Fluor™ 488; Thermo Fisher Scientific, #A-21121, Waltham, MA, USA) was applied (1:200 in 0.1% Tween-20/TBS + 1% BSA) for 1 h at RT in the dark. Following further washing, nuclei were counterstained with DAPI (4′,6-diamidino-2-phenylindole, CyStain^®^, 1:10, UV Ploidy, Sysmex Partec GmbH, Görlitz, Germany) for 5 min, and slides were mounted with Mowiol^®^ (Mowiol^®^ 4-88, Carl Roth GmbH + Co. KG, Karlsruhe, Germany). Coverslips were applied (Menzel Deckgläser, 24 × 60 mm, Gerhard Menzel GmbH, Leverkusen, Germany) (The full staining protocol is provided in Supplementary Table S2).

Fluorescence imaging was performed using the Thunder Imager system (DMi8 microscope with DFC9000 GTC sCMOS camera, LAS X software version number 5.3.1, Leica, Wetzlar, Germany). Images (Objectives: HC PL APO 20×/0.4 and 40×/0.95 DRY) were acquired with LAS X Software, Leica. Thresholding based on negative controls was used to detect specific immunostaining. For confocal imaging, confocal z-stacks were acquired on a Zeiss LSM 800 (Carl Zeiss, Göttingen, Germany) fitted with a 405/488/561/640 nm laser module and an Airyscan detector. Z-stacks were collected at 170 nm increments. To ensure comparability for intensity quantification, all images were captured using identical acquisition parameters in ZEN 3.4 software (Carl Zeiss, Göttingen, Germany) and processed with the same Airyscan settings. Images were acquired with a 63×/1.40 oil objective at 16-bit depth, with a pixel scale of 0.032 μm (field size 77.24 μm). Dual tracks (488 nm and 405 nm) were recorded at low laser power (0.05%) with optimized pinhole and super-resolution settings. White-balance parameters were optimized for each antibody–fluorophore combination and then uniformly applied to all corresponding samples. As a control for non-specific secondary antibody binding, secondary antibodies were applied to parallel samples under each experimental condition in the absence of primary antibodies [40].

### 2.7. Statistical Methods

Data are presented as mean ± standard error of the mean (SEM). Unless otherwise stated, “***n***” refers to the number of animals (*n* = 6). Statistical analyses were conducted using GraphPad Prism software (Version 10.4.1 (627), 2024, GraphPad Software, Inc., San Diego, CA, USA) for Windows^®^ 64-bit. Data distribution was assessed using the Shapiro–Wilk normality test. For comparisons between two groups, normally distributed data were analyzed using Student’s *t*-test (unpaired), while non-normally distributed data were evaluated using the Mann–Whitney *U* test. For comparisons involving more than two groups, one-way analysis of variance (ANOVA) followed by Tukey’s post hoc test was used for normally distributed data, and non-parametric comparisons were performed using the Kruskal–Wallis *H* test followed by Dunn’s post hoc test. Histomorphometry, goblet cell distribution, and mucin gel layer thickness measurements were analyzed accordingly. A *p*-value of <0.05 was considered statistically significant; values between 0.05 and 0.10 were interpreted as trends. Significance levels in figures were indicated as follows: *ns* (not significant, *p* ≥ 0.05), * (*p* < 0.05), ** (*p* < 0.01), *** (*p* < 0.001).

### 2.8. Figure Preparation

Two schematic illustrations (Figures 3A and 9E) were created with software assistance. ChatGPT-5 (o4-mini-high, OpenAI; accessed 1 August 2025) was used to refine the visual layout and labeling, and Illustrae (illustrae.co; accessed 4 August 2025) was used to generate and edit the final vector artwork from author-provided prompts. The authors verified the scientific accuracy of all elements.

## 3. Results

### 3.1. Histomorphometry–Villi in Jejunum and Ileum

**Figure 2:**
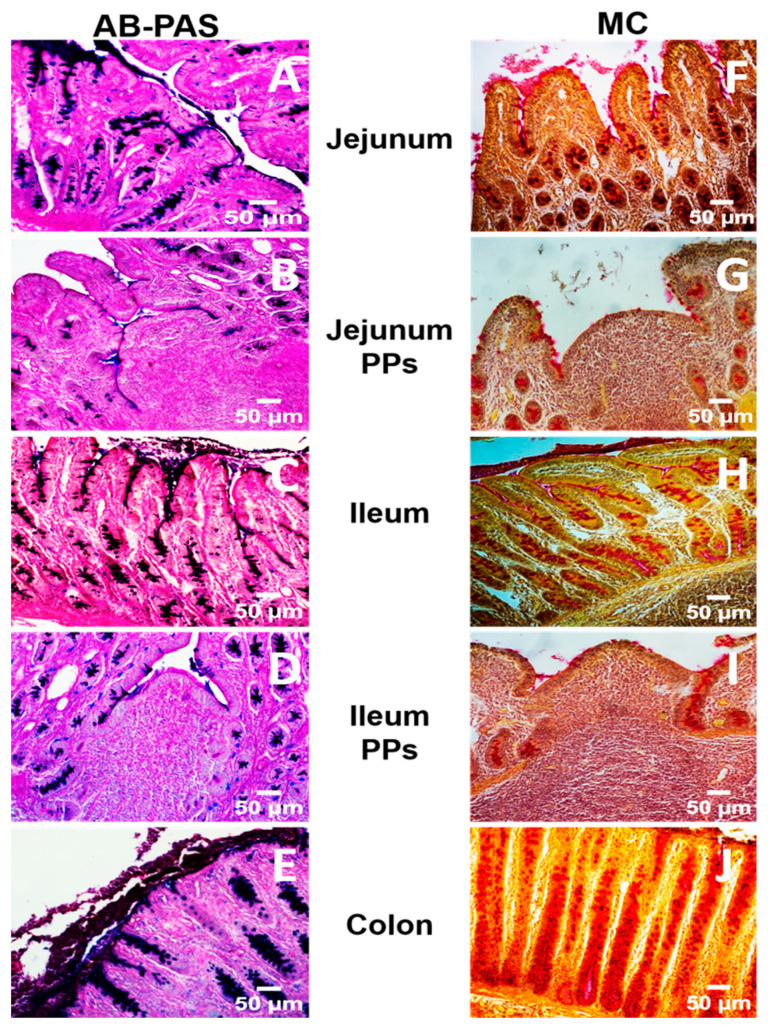
Representative histological micrographs of jejunum and ileum without and with Peyer’s patches (PPs), and colon stained with Alcian Blue–Periodic Acid–Schiff (AB-PAS) (**A**–**E**) and Mucicarmine (MC) (**F**–**J**). Histomorphometric analysis of follicle-associated epithelium, villi, and colonic crypts was conducted in the jejunum, ileum, and colon using two staining methods. (*Scale bar* = 50 μm).

Across both jejunal and ileal segments, morphometric indices obtained with AB-PAS and MC stains were not statistically different (*p* > 0.05). The 95% confidence intervals overlapped substantially, and differences fell within single standard deviation (Table 1 and Figure 2).

### 3.2. Follicle-Associated Epithelium (FAE) in Jejunum and Ileum Are Similar in Length

In the jejunum, the FAE length measured 251 ± 29 μm with AB-PAS staining and 230 ± 17 μm with MC staining. In the ileum, the corresponding lengths were 293 ± 33 μm (AB-PAS) and 244 ± 17 μm (MC). The FAE length in PPs was therefore similar between the ileum and jejunum, regardless of the staining method.

### 3.3. Comparison of Mucin Gel Layer Thickness Across All Evaluated Intestinal Segments

**Figure 3:**
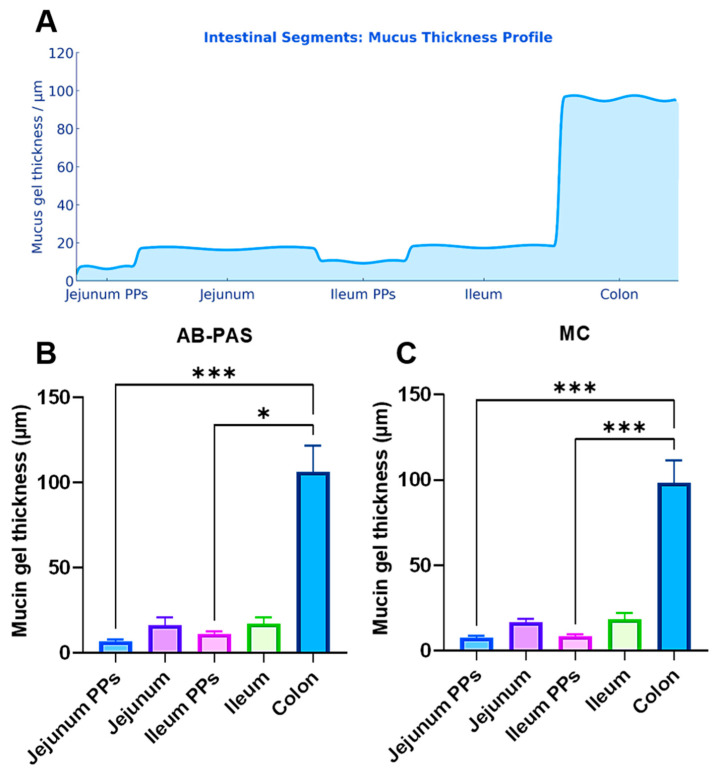
(**A**) Proximal-to-distal gradient of mucus-gel thickness in the porcine intestine. The schema shows a stepwise increase in mucus thickness from jejunum to colon. Over the follicle-associated epithelium (FAE) of Peyer’s patches (PPs) in both jejunum and ileum, the mucus layer is thinnest (around 10 μm). In adjacent mucosa, thickness increases progressively along the small intestine, exceeding ~17 μm in the distal ileum. A further step-increase occurs at the ileo–colonic junction, and the colon exhibits the greatest thickness (~100 μm). Alcian Blue–Periodic Acid–Schiff (AB-PAS) and mucicarmine (MC) stains supports the marked thinning over PPs and the proximal-to-distal thickening of the mucus barrier. (**B**,**C**) Thickness of the mucus layer (mean ± SEM) measured on the surface PPs in the jejunum and ileum, and on the adjacent regular mucosa of the jejunum, ileum, and colon, as determined by AB-PAS and MC (Kruskal–Wallis *H* test followed by Dunn’s post hoc test for AB-PAS and one-way analysis of variance (ANOVA) followed by Tukey’s post hoc test for MC). (*n* = 6; ns *p* ≥ 0.05; * *p* < 0.05; ** *p* < 0.01; *** *p* < 0.001).

Across all evaluated intestinal segments (jejunum, ileum, and colon), AB-PAS and MC staining yielded comparable mucin thickness measurements, with no stain-related differences detected. Over Peyer’s patches, the mucin layer remained consistently thin (<12 μm). By contrast, in the regular mucosa a pronounced proximal-to-distal gradient was evident, with mucin thickness increasing modestly from the jejunum to the ileum (~1.1-fold) and then markedly from the ileum to the colon (~6-fold) (Table 2 and Figure 3).

### 3.4. Quantification of GCs Using the AB-PAS Staining Technique

**Figure 4:**
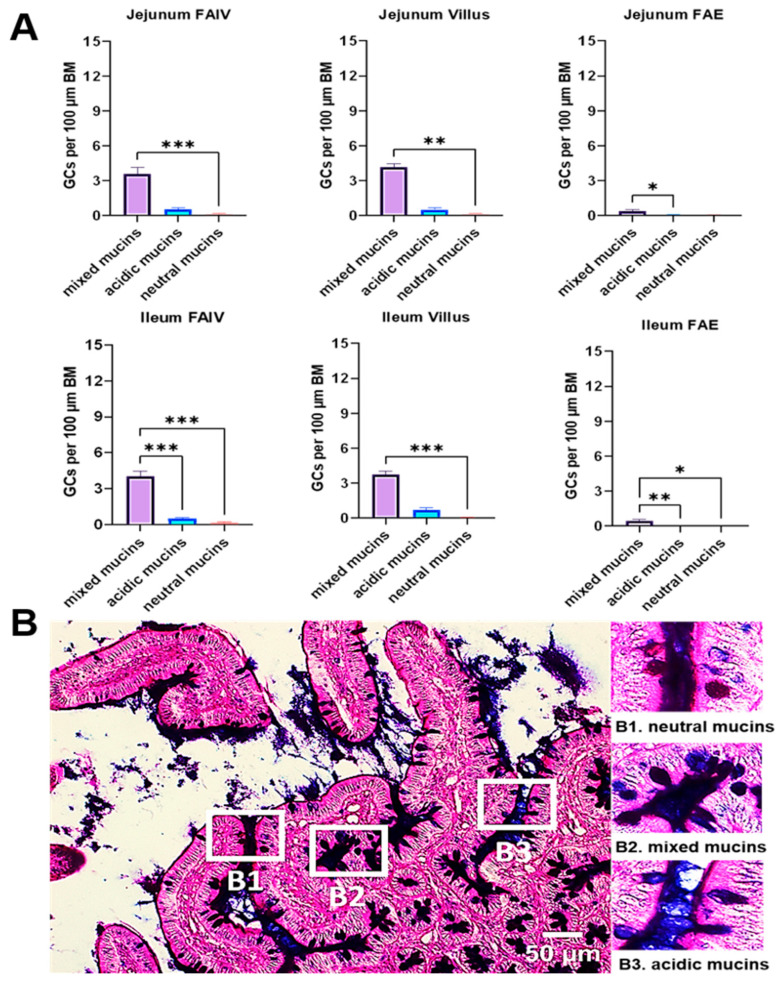
(**A**) Relative frequency of goblet cells (GCs)-number per 100 μm of basement membrane (BM)-by mucin type (neutral, mixed, acidic) from AB-PAS staining. Goblet cell counts in FAIV versus adjacent regular villi of jejunum and ileum, and specifically within the FAE overlying PPs (after Shapiro–Wilk normality assessment, all comparisons were analyzed with Kruskal–Wallis *H* test followed by Dunn’s post hoc test; only FAIV in ileum was analyzed with ANOVA followed by Tukey’s post hoc test). (*n* = 6; ns *p* ≥ 0.05; * *p* < 0.05; ** *p* < 0.01; *** *p* < 0.001). (**B**) Representative AB-PAS-stained jejunum section showing neutral mucins **B1** (magenta; AB−, PAS+), mixed mucins **B2** (deep purple; AB+ PAS+), and acidic mucins **B3** (blue; AB+ PAS-). (*Scale Bar* = 50 μm). Abbreviations: FAIV, follicle-associated intestinal villus; AB-PAS, Alcian Blue–Periodic Acid–Schiff; FAE, follicle-associated epithelium, PP, Peyer’s patch.

The primary advantage of the AB-PAS staining method is its capacity to differentiate and quantify distinct mucin types within the porcine small intestine. Across all evaluated segments—FAIV in jejunum and ileum, regular villi in jejunum and ileum, and FAE covering PPs in both jejunum and ileum—a consistent distribution pattern of mucin types was observed. Mixed mucins were the predominant type, followed by acidic and neutral mucins. Mean values representing the relative frequency of GCs per 100 μm of basement membrane (BM) are summarized in Figure 4.

### 3.5. Comparison of Goblet Cell Counts Across Intestinal Segments Using Both Staining Methods

**Figure 5:**
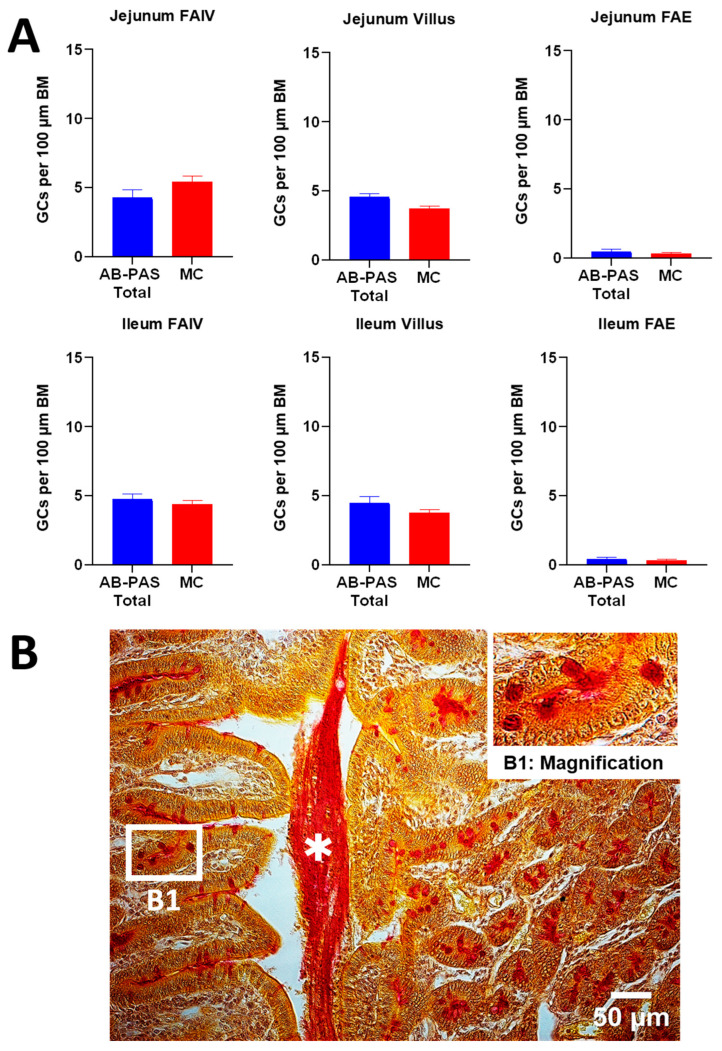
(**A**) Goblet cell density (GCs per 100 μm basement membrane (BM)) on FAIV versus adjacent villi in jejunum and ileum, and on FAE in jejunum and ileum, using AB-PAS and MC staining. All of the data were normally distributed and compared by Student’s *t*-test. (*n* = 6; ns *p* ≥ 0.05; * *p* < 0.05; ** *p* < 0.01; *** *p* < 0.001). (**B**) Mucicarmine-stained ileal section with a condensed mucus layer partially peeled from the right villus apices (middle; asterisk). The upper-right inset (**B1**) magnifies the boxed area and highlights goblet cells whose mucins range from pink to deep red. (*Scale Bar* = 50 μm). Abbreviations: FAIV, follicle-associated intestinal villus; FAE, follicle-associated epithelium; AB-PAS, Alcian Blue–Periodic Acid–Schiff; MC, mucicarmine.

We further compared the total relative frequency of GCs per 100 μm of basement membrane, obtained using the two staining methods (AB-PAS and MC) across identical intestinal segments. No statistically significant differences in total relative goblet cell counts were detected between staining methods across the FAE, FAIV, regular villi and colonic segments (Figure 5).

### 3.6. Comparative Assessment of Goblet Cell Distribution Shows Reduced Numbers in the FAE

**Figure 6:**
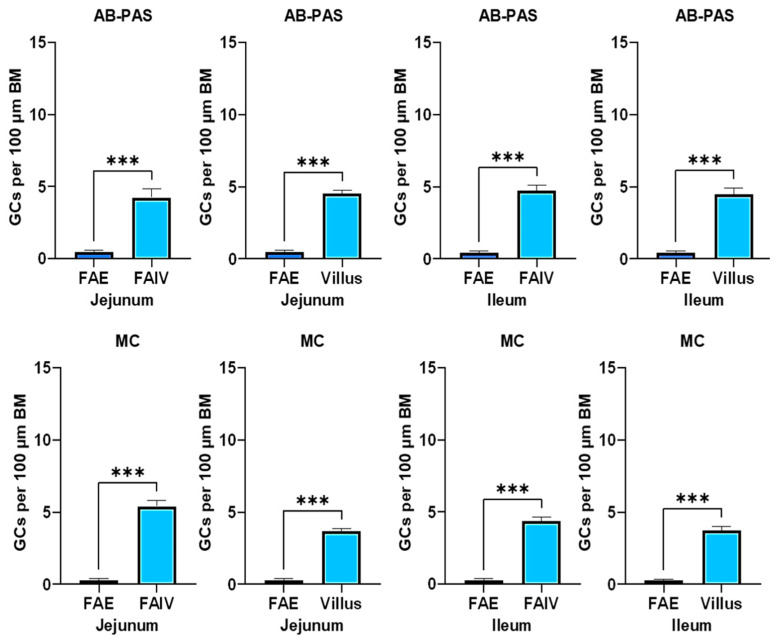
Comparison of the total relative number of goblets cells (GCs) per 100 μm of basement membrane (BM) overlying the FAE with either the follicle-associated villus (FAIV) or regular villi in the jejunum and ileum, using both staining methods. All the data were normally distributed and analyzed using Student’s *t*-test. (*n* = 6; ns *p* ≥ 0.05; * *p* < 0.05; ** *p* < 0.01; *** *p* < 0.001). Abbreviations: FAIV, follicle-associated intestinal villus; FAE, follicle-associated epithelium; AB-PAS, Alcian Blue–Periodic Acid–Schiff; MC, mucicarmine.

We analyzed the total relative number of GCs per 100 μm of basement membrane (BM) overlying the FAE, the FAIV, and regular villi in both the jejunum and ileum. A statistically significant reduction in goblet cell numbers was observed in the FAE compared to the FAIV, independent of staining method. Similarly, when comparing the FAE to regular villi, a comparable decrease in goblet cell density was evident in both small intestinal segments. No statistically significant differences were found between FAIV and regular villi in either the jejunum or ileum—indicating comparable goblet-cell density—except with mucicarmine staining, where FAIV in the jejunum differed significantly from regular jejunal villi (see Supplementary Figure S3) (Figure 6).

### 3.7. Colon

**Figure 7:**
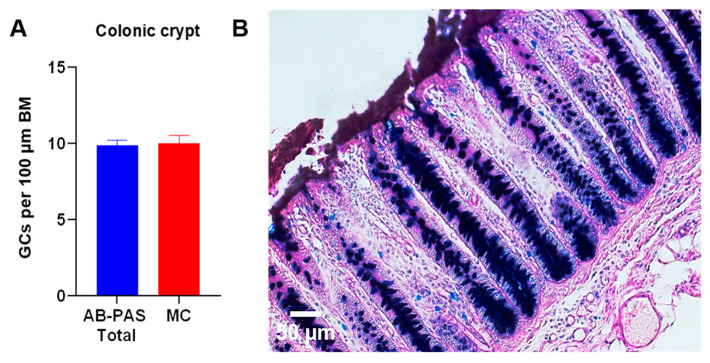
(**A**) Goblet cell density (GCs per 100 μm basement membrane (BM)) in colonic crypts using AB-PAS and MC staining. Quantification confirmed that the colon has the highest goblet cell frequency per 100 μm of BM. The non-normally distributed data were compared using Mann–Whitney *U* test. (*n* = 6; ns *p* ≥ 0.05). (**B**) Histological sections demonstrated that GCs in the base of the colonic crypts contain mixed mucins apically and acidic mucins basally. (*Scale bar* = 50 μm.) Abbreviations: GCs, goblet cells; AB-PAS, Alcian Blue–Periodic Acid–Schiff; MC, mucicarmine.

In the colon, the measurement of the mucin gel layer (Figure 3) and the goblet cell density within colonic crypts (Figure 7) demonstrated that the colon exhibits the highest goblet cell number per 100 μm of basement membrane (BM) and the thickest mucin layer in comparison to the small intestine. The morphometric analysis of the crypt architecture showed a crypt depth of 375 ± 17 μm using AB-PAS staining and 377 ± 19 μm in MC staining. This indicates a consistent structural dimension independent of the staining methods. Notably, AB-PAS-stained histological sections of porcine colon revealed that GCs exclusively contain mixed mucins within their apical regions of the cell, whereas acidic mucins predominated in the basal cellular portions, no neutral mucins were noticed (Figure 7B).

### 3.8. Fluorescence Microscopy Analysis of CK18^+^ GCs in Colonic Epithelium

**Figure 8:**
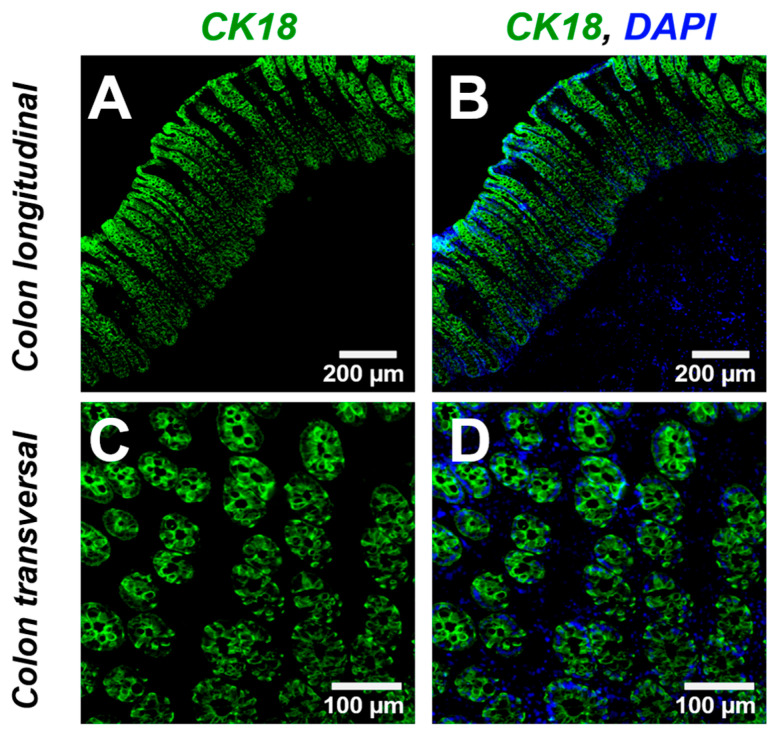
Immunofluorescence micrographs of CK18-expressing GCs within colonic crypts. The upper panel (**A**,**B**) shows longitudinal sections, in which CK18^+^ GCs are prominently distributed along the crypt axis. The lower panel (**C**,**D**) displays high-magnification transverse sections, revealing GCs radially arranged around the crypt lumen. These cells exhibit apically located intracellular cavities devoid of CK18 immunoreactivity, consistent with mucin granule accumulation sites. (*Scale bars:* upper panel = 200 μm, lower panel = 100 μm). Abbreviations: GCs, goblet cells; CK18, cytokeratin-18 (green); panel B and D: Colocalization of CK18 and nuclei (DAPI, blue).

The anti-CK18 immunofluorescence staining showed a positive labeling in all GCs. Immunofluorescence analysis revealed strong CK18 expression within the colonic mucosa, evident in both transverse and longitudinal sections of the colonic crypts. No CK18 immunoreactivity was detected in the submucosa or *muscularis* layers (Figure 8).

### 3.9. Confocal Microscopy Imaging of Solitary CK18^+^ GCs in Jejunal PPs and Colon

**Figure 9:**
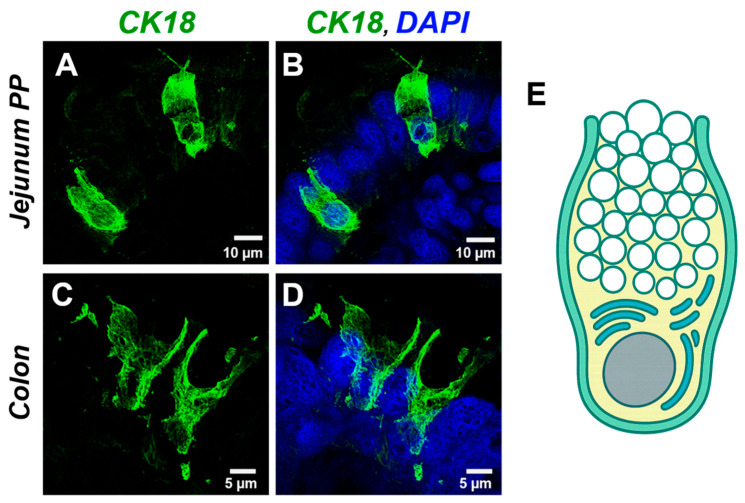
Confocal micrographs show that the theca of GCs is partially composed of CK18, contributing to the structural integrity necessary for sustained secretory activity. Representative images display two CK18^+^ GCs located along the FAE of the jejunal PPs in the upper panel (**A**,**B**) and two CK18^+^ GCs from colonic mucosa in the lower panel (**C**,**D**) (*Scale bars:* upper panel = 10 μm; lower panel = 5 μm, CK18, cytokeratin-18 (green); nuclei, DAPI (blue)). Illustration in (**E**) represents a longitudinal section of a goblet cell. The apical domain is distended and directed to the lumen, with active secretion of mucin granules, visualized as densely packed white vesicles. The basal region narrows into a stem-like segment housing the basally positioned nucleus and biosynthetic organelles, including the endoplasmic reticulum and Golgi apparatus, essential for mucin production. Abbreviations: GCs, goblet cells; PP, Peyer’s patch; panel (**B**,**D**): Colocalization of CK18 (green) and nuclei (DAPI, blue).

Confocal microscopy analysis of CK18^+^ GCs in both jejunal PPs and the colon revealed the characteristic goblet or cup-shaped morphology. The lateral domain of the cell is reinforced by a prominent theca, composed in part of densely packed intermediate filaments. Apical and medial to this cytoskeletal structure, a central cellular cavity is observed, within which mucin granules are tightly packed. The basal region of the cell, resembling a stem-like structure, houses cellular organelles and the nucleus, the latter clearly delineated by DAPI staining. The basal end of the cell is firmly anchored to the basement membrane (Figure 9).

## 4. Discussion

The thickness of the gastrointestinal mucus layer reflects a dynamic balance between mucin synthesis and secretion by GCs and its loss through bacterial enzymatic degradation, chemical dissolution, and mechanical shear—processes that are especially pronounced in distal intestinal segments [41,42,43]. Numerous in vitro and in vivo studies have investigated the thickness of the mucin gel layer across the GIT in animal models and humans. In vitro assessments have been conducted in rats [44,45], frogs [44], and humans [44], while in vivo measurements have been performed primarily in rats [46,47,48] and humans [49,50]. However, variation in methods (e.g., fixation protocols and measurement techniques) and in anatomical focus has led to inconsistent findings across studies [51]. For example, several studies report a thicker mucus gel layer in the stomach than in the cecum and colon in both humans and rats [43,46,47]. Conversely, Atuma et al. reported greater mucus thickness in the rat colon, underscoring the variability among studies [14]. These inconsistencies highlight the limits of extrapolating rodent data to the human GIT. Although rats and mice are invaluable for basic gastrointestinal biology, they differ substantially from humans in anatomy, physiology, and biochemistry—including a simpler stomach, faster intestinal transit, and distinct patterns of mucin glycosylation and goblet-cell distribution [52]. Large-animal models such as pigs offer a more appropriate alternative because of their close anatomical and physiological resemblance to humans. The porcine gastrointestinal tract mirrors the human system in mucosal architecture, peristalsis, mucin biochemistry, and the distribution of immune tissues. At the same time, species-specific differences—in luminal pH profiles, bile and pancreatic secretions, gastrointestinal fluid volumes, microbiota, and propulsive activity—can affect dissolution, transit, and uptake. Transit is also age dependent in pigs, especially around weaning. Accordingly, physiological stage and regional conditions should be matched when extrapolating porcine findings to humans, clarifying both the model’s translational relevance and its limits [52,53]. To date, no study has comprehensively measured mucin gel-layer thickness in porcine intestine using histology on mucin-preserving Carnoy’s–fixed tissues, nor compared goblet-cell distribution along the intestinal axis—particularly across the follicle-associated epithelium (FAE) overlying Peyer’s patches (PPs)—using dual histochemical stains. Moreover, the presence and functional significance of a mucus layer over the FAE remain poorly defined, and there is no consensus on the frequency or functional state of GCs within this specialized epithelium [54,55,56].

### 4.1. The Mucin Gel Layer

Atuma et al. used an in vivo glass-micropipette technique in anesthetized rats to quantify the thickness of the gastrointestinal mucus gel layer [14]. Atuma et al. reported mean mucus thicknesses of 189 ± 11 μm (gastric corpus), 274 ± 41 μm (gastric antrum), 170 ± 38 μm (duodenum), 123 ± 4 μm (jejunum), 480 ± 47 μm (ileum), and 830 ± 110 μm (colon). By distinguishing loosely adherent from firmly adherent layers, they provided a more comprehensive view of mucus-barrier architecture. Their data indicate a continuous, robust mucus layer throughout the GIT, substantially thicker than in earlier reports. In a separate in vivo study, Strugala et al. measured proximal colonic mucus thickness in anesthetized rats, reporting values from 213 to 1486 μm (mean 642 ± 54.8 μm; *n* = 34), further underscoring the dynamic variability of the mucus barrier in rodent models [57]. Our regional patterns align with pig data from Varum et al. Using 10-μm cryosections stained with a modified PAS/Alcian Blue protocol, they reported secreted mucus-gel thicknesses (mean ± SD, μm) as follows: stomach—fundus 190.7 ± 80.7, body 213.9 ± 87.9, antrum 222.2 ± 112.2; small intestine—duodenum 25.6 ± 12.2, jejunum 35.3 ± 17.8, ileum 53.8 ± 22.1; large intestine—cecum 37.2 ± 16.1, ascending colon 68.1 ± 36.5, transverse colon 83.6 ± 36.2, descending colon 76.3 ± 56.7, rectum 58.8 ± 27.9—showing a proximal-to-distal increase that mirrors our gradient. Because methodology strongly affects absolute values (e.g., cryosections vs. Carnoy-fixed paraffin sections can yield several-fold differences), cross-study comparisons should emphasize regional patterns rather than raw measurements [58]. Using an inverted phase-contrast microscope with a calibrated eyepiece, Pullan et al. measured adherent colonic mucus-gel thickness in fresh human surgical specimens, reporting control values (mean ± SD) of 107 ± 48 μm in the right colon, 134 ± 68 μm in the left colon, and 155 ± 54 μm in the rectum. Relative to this benchmark, our colonic measurements overlap the human ranges, supporting external validity while acknowledging species- and method-dependent differences [59]. In our comparative analysis of porcine intestinal segments, we observed pronounced regional variation in mucus layer thickness, with a reduction on the FAE of PPs. However, a continuous but diminished mucin stratum remains present in both jejunal and ileal PPs. This finding supports the concept of localized mucus modulation in regions of immunological specialization and further emphasizes the need for high-resolution mapping of mucus distribution in large-animal models. Ermund et al. demonstrated that murine Peyer’s patches are covered by a distinct mucus layer [17,28]. Using bacterium-sized fluorescent beads, they showed that this layer is permeable to particulates: beads progressively penetrated the mucus and accumulated within and immediately above the FAE. With its reduced density and thickness, the PP-associated mucus retains sufficient penetrability to facilitate luminal antigen sampling. They also noted an absence of spontaneous mucus secretion over PPs, in contrast to adjacent villous epithelium. Upon stimulation with carbachol and prostaglandin E_2_, secretion was observed from surrounding follicle-associated intestinal villi (FAIV) but not from the FAE itself. Although MUC2-positive goblet cells are present within the FAE, active secretion was not detected even 40 min after stimulation. These findings suggest functional divergence of FAE-localized goblet cells and support the hypothesis that a subset may perform non-secretory roles, such as antigen delivery via goblet cell-associated antigen passages (GAPs) [60]. In our porcine samples, the barrier over Peyer’s patches is reduced but not absent: a continuous mucin layer spans the FAE, yet it is markedly thinner (~7–11 μm) than over adjacent villi (16–18 μm) and far thinner than colonic mucus (~100 μm). The FAE also contains fewer goblet cells than neighboring FAIV and regular villi. We interpret this configuration as an adaptation that lowers resistance to controlled antigen uptake—buffered by local safeguards (sIgA, antimicrobial effectors, and compensatory FAIV mucin secretion)—while acknowledging that M cell specialization and a thinner interface introduce potential vulnerability. Overall, the FAE appears to be a finely tuned balance between sampling efficiency and infection risk. Quantitative histology by Onori et al. in rat ileal Peyer’s patches confirms the low frequency of goblet cells within the FAE, declining from 8.57 ± 1.72% basally to 3.14 ± 2.48% apically [55]. Importantly, species-specific variation in goblet-cell density within lymphoid-associated epithelia has also been reported in lagomorphs. Beyaz et al. reported an absence of goblet cells within Peyer’s patches of Angora rabbits, although some isolated lymphoid follicles contained sparse goblet cells. These follicles, encircled by FAE, lacked clear differentiation into germinal centers or interfollicular regions, underscoring interspecies variability in mucosal immune architecture [61].

### 4.2. The Goblet Cells

The colonic mucus gel layer is generated by mucin secretion from two goblet-cell populations: crypt-resident GCs and intercrypt goblet cells (icGCs) in the surface epithelium [62]. Functional studies indicate that mucus from crypt GCs forms a dense, laminated barrier against bacteria and microparticles, whereas icGC-derived mucus remains penetrable to small particles yet still restricts bacterial access [62]. Specian et al. provided an ultrastructural description of mature, mucin-secreting goblet cells (GCs) at villus tips and in intercrypt regions of the colon. Hallmark secretory features included a basal ovoid nucleus surrounded by mitochondria and rough endoplasmic reticulum (rER), a supranuclear Golgi apparatus, and an apical domain packed with mucin granules enclosed by a cytoskeletal theca. This theca—composed of circumferential and longitudinal intermediate filaments with spiral reinforcements—maintains cell architecture, while underlying microtubules support intracellular organization [63].

Using immunofluorescence for cytokeratin 18 (CK18)—a principal epithelial intermediate-filament protein—we confirm and extend these structural insights. Unlike earlier reports that restricted CK18 expression to porcine M cells, we observed robust CK18 signal in the GCs [29]. We observed strong CK18 expression in goblet cells throughout the porcine small and large intestines. In confocal micrographs, the theca appeared as a CK18-positive cytoskeletal scaffold partially encasing apical cavities filled with mucin granules; its distension around accumulated mucin is consistent with a mature secretory profile. Over the past decade, the traditional view of goblet cells as passive mucin secretors has shifted—GCs are now recognized as active participants in immune surveillance and epithelial signaling [62,64]. Beyond forming the mucus barrier, GCs regulate mucosal immunity by modulating innate responses and facilitating antigen sampling that primes adaptive immune activation. A paradigm-shifting study by Nyström et al. identified two goblet-cell lineages: a canonical lineage that produces gel-forming mucus and a noncanonical, enterocyte-like lineage that likely supports luminal sensing and local immune regulation. A distinct subset—sentinel goblet cells (senGCs) at the mouths of distal colonic crypts—acts as gatekeepers in humans and mice, coordinating protective responses to luminal antigens [64]. Consistent with observations over Peyer’s patches, Ermund et al. reported that the FAE) contains MUC2-positive goblet cells but exhibits minimal stimulus-evoked secretion [17,28]. Together with the reduced goblet-cell numbers, this pattern suggests an enrichment of less secretory, possibly noncanonical phenotypes and warrants further investigation.

### 4.3. Histochemical Techniques

In an early mucin histochemistry study, Matsuo et al. used Carnoy’s fixative to preserve mucins in human colonic tissue and demonstrated AB–PAS-positive (blue/purple) goblet cells, consistent with mixed or acidic mucin profiles within single cells [35]. We observed a similar staining pattern in porcine colonic crypts, reinforcing biochemical and morphological parallels with the human GIT. AB–PAS staining identified mixed, acidic, and neutral mucins throughout the porcine GIT, with mixed mucins predominating across regions. This distribution aligns with Law et al., who observed a predominance of purple-stained goblet cells in AB–PAS-stained sections of young piglets, indicating mixed neutral and acidic mucins [65]. Law et al. also showed that mixed mucin-containing GCs were concentrated in the upper crypts and lower villi of the small intestine, whereas acidic mucin-containing GCs were more frequent in the deeper crypts and lower villi [65]. In the colon, acidic mucins were increased, although most goblet cells still exhibited mixed mucins. Despite extensive histological characterization, the functional significance of regional mucin heterogeneity remains incompletely understood [66]. The physiological roles of distinct mucin subtypes—particularly in antigen sampling, host–microbe interactions, and barrier function—require further study, especially within anatomically and immunologically specialized niches such as the FAE over PPs.

### 4.4. Methodological Optimization for GCs and Mucin Visualization in Porcine Intestinal Tissue

One objective was to establish a robust histochemical workflow for goblet-cell visualization and quantitative measurement of the mucin gel layer across porcine intestinal segments. We achieved this using two stains—Alcian Blue–Periodic Acid–Schiff (AB–PAS) and Mucicarmine—each with complementary strengths. Across the gastrointestinal axis, the methods showed no significant differences in goblet-cell density or distribution, or in mucin-layer thickness, supporting their use in routine histology. Reliable in vitro and ex vivo assessment of mucins requires appropriate fixation; Carnoy’s solution, a nonaqueous, ethanol-based fixative, provided higher preservation of mucin architecture. As emphasized by Röhe et al., fixation must preserve the native configuration and localization of mucus to ensure histological validity. Beyond Carnoy’s solution, alternative approaches—including cryopreservation, methacarn, and buffered paraformaldehyde—have been evaluated for mucin preservation. Röhe and colleagues showed that rapid freezing in liquid nitrogen, followed by chemical post-fixation and AB–PAS staining, enables accurate measurement of porcine colonic mucus thickness [39]. Each staining method has strengths and limitations. Owing to its simplicity, speed, and low cost, mucicarmine (MC) is suitable for laboratories without specialized histology equipment. However, MC labels all mucins irrespective of charge or metabolic profile and cannot distinguish mucin subtypes. In addition, the strong pink coloration produced by MC with Metanil Yellow can hinder reliable manual counting of GCs under brightfield microscopy; automated histomorphometry and digital image analysis may mitigate this issue. By contrast, Alcian Blue–Periodic Acid–Schiff (AB–PAS) differentiates mucin subtypes but requires greater technical proficiency and longer processing times. In combination, Alcian Blue and Schiff reagent yield blue staining for acidic mucins, magenta for neutral mucins, and purple for mixed mucins. This provides informative biochemical and spatial data—particularly relevant for studies of regional mucosa—and, compared with MC, affords better contrast between GCs and background tissue, enabling more precise cell identification and quantification. Building on our findings, we propose that Alcian Blue–Periodic acid–Schiff (AB-PAS) and mucicarmine staining of human tissue samples could become an adjunctive tool in colorectal pathology. We hypothesize that these methods can provide help by addressing diagnostic ambiguity left by hematoxylin–eosin (H&E) staining, thereby enabling a more definitive characterization of tumor mucin. This approach could not only support the accurate subtyping of mucin-producing adenocarcinomas, including signet-ring cell variants, but also offer a reliable method for pathologists to objectively meet the World Health Organization (WHO) criterion for mucinous carcinoma (>50% extracellular mucin), potentially leading to more precise diagnoses and better-informed clinical decisions [67,68].

### 4.5. Study Limitations

Because this is an ex vivo, histology study, dynamic mucin secretion and physiological shear are not captured. Pre-fixation handling (rinsing and mesenteric opening) and the requirement for a continuous ≥100-μm mucus stretch may bias thickness estimates toward better-preserved regions with finer preserved outer mucus layer. Although Carnoy’s fixative preserves mucus well, subsequent ethanol/xylene processing and 5-μm sectioning can still introduce shrinkage or redistribution artifacts.

Finally, goblet-cell counts normalized per 100 μm of basement membrane do not account for three-dimensional epithelial thickness or villus density. Future work should delineate the roles of mucins and goblet cells under more realistic physiological conditions.

## 5. Conclusions

This study maps regional mucus-layer thickness and goblet-cell distribution along the porcine intestine, with emphasis on jejunal and ileal Peyer’s patches. Carnoy’s fixation preserved mucus architecture, and AB–PAS and mucicarmine provided largely concordant staining. Peyer’s patches showed a markedly thinner mucus layer and fewer goblet cells than adjacent villi and colon and CK18 was consistently expressed in goblet cells across small and large intestines, supporting its use as a marker.

## Figures and Tables

**Table 1:** Summary of villus morphometry (μm, mean ± SEM) for jejunum and ileum (*n = 6*).

Segment	Staining Method	Villus Height (μm)	Villus Width (μm)
**Jejunum**	**AB-PAS**	217 ± 22	90 ± 6
	**MC**	229 ± 24	91 ± 7
**Ileum**	**AB-PAS**	212 ± 38	119 ± 35
	**MC**	197 ± 25	100 ± 7

**Table 2:** Mean mucin layer thickness (μm, mean ± SEM) by segment (*n = 6*).

Segment	Measurement Site	AB-PAS (μm)	MC (μm)
**Jejunum**	**Peyer’s patch**	7 ± 1.1	8 ± 0.9
**Ileum**	**Peyer’s patch**	11 ± 1.7	8.6 ± 0.8
**Jejunum**	**Regular intestinal mucosa**	16 ± 4.7	17 ± 1.6
**Ileum**	**Regular intestinal mucosa**	17 ± 3.6	18 ± 3.6
**Colon**	**Mucosa**	106 ± 15	98 ± 13

## Data Availability

The complete raw data are available upon request.

## Supplementary Materials

The following supporting information can be downloaded at: https://www.mdpi.com/article/10.3390/ani15192852/s1, Table S1: Modified staining protocols, AB-PAS and MC, used in our study to identify mucosubstances and goblet cells (GCs); Figure S1: Porcine jejunum—luminal mucus gel layer. (A) Alcian Blue–Periodic Acid–Schiff (AB–PAS); (B) Mucicarmine. Both stains highlight the luminal mucus gel layer (asterisks) overlying intact villous epithelium, showing the characteristic small-intestinal pattern: the layer is comparatively thin, irregular, and concentrated at villus tips, and may be discontinuous between adjacent villi. In AB–PAS (A), goblet-cell mucins within the epithelium appear purple and the surface gel forms a dark-purple luminal coating; in mucicarmine (B), the gel appears as a dense deep-red band draped over villus tips with uneven thickness. Physiologically, the jejunal mucus forms a single, loose, MUC2-based gel that partially fills intervillous spaces and usually caps villus tips; and thinner/more heterogeneous than colonic mucus. (Scale Bar = 50 μm); Figure S2: Ileum (mucicarmine)—measurement of the mucus gel layer. Light micrograph of porcine small-intestinal mucosa (ileal villus) stained with mucicarmine. The luminal mucus gel layer appears as a deep-red band and is marked with an asterisk (left), overlying intact villous epithelium. Thickness was measured in ImageJ using three adjacent, perpendicular line profiles (white) drawn from the epithelial surface into the mucus and spaced 10 μm apart (yellow label). The corresponding measurements are shown next to each profile: 22.3 μm (1), 15.2 μm (2), and 17.2 μm (3). The field meets the pre-specified inclusion criterion of a continuous mucus layer ≥ 100 μm in length along intact epithelium, illustrated by the 100 μm reference ruler at the top. The ImageJ “Results” table at the bottom documents the measurement outputs and metadata (*Scale Bar* = 50 μm); Table S2: Cytokeratin 18 (CK18) indirect immunofluorescence staining protocol; Figure S3: Comparison of the total relative number of goblet cells (GCs) per 100 μm of basement membrane (BM) between the FAIV and regular villi in either the jejunum or ileum. All the data were normally distributed and analyzed using Student’s *t*-test. (*n* = 6; ns *p* ≥ 0.05; * *p* < 0.05; ** *p* < 0.01; *** *p* < 0.001). Abbreviations: FAIV, follicle-associated intestinal villus; AB-PAS, Alcian Blue–Periodic Acid–Schiff; MC, mucicarmine.

## Abbreviations

The following abbreviations are used in this manuscript:

AB	Alcian Blue
AB-PAS	Alcian Blue–Periodic Acid–Schiff
BM	Basement membrane
BW	Body weight
CK18	Cytokeratin 18
DA	Dome area
DAPI	4′,6-diamidino-2-phenylindole
FAE	Follicle-associated epithelium
FAIC	Follicle-associated intestinal crypt
FAIV	Follicle-associated intestinal villus
GALT	Gut-associated lymphoid tissue
GAPs	Goblet cell-associated antigen passages
GC	Germinal center
GCs	Goblet cells
GIT	Gastrointestinal tract
gIC	gram of intestinal content
icGCs	Intercrypt goblet cells
IgA	Immunoglobulin A
IF	Immunofluorescence
MC	Mucicarmine
M cells	Microfold cells
MUC	“mucin” (the mucin gene/protein family)
PAS	Periodic Acid–Schiff
pIgR	Polymeric immunoglobulin receptor
PPs	Peyer’s patches
rER	Rough endoplasmic reticulum
RT	Room temperature
sIgA	Secretory immunoglobulin A
SD	Standard Deviation
SED	Subepithelial dome
senGCs	Sentinel goblet cells
SEM	Standard error of the mean
TBS	Tris-buffered saline
16S rRNA	16S ribosomal RNA
