# Supplementary material for: Goblet Cells and Mucus Composition in Jejunum and Ileum Containing Peyer’s Patches and in Colon: A Study in Pigs
Source: Animals (Basel). 2025 Sep 29;15(19):2852. doi: 10.3390/ani15192852 (PMC12524252; doi:10.3390/ani15192852)
Supplement: Supplementary file 1 [file animals-15-02852-s001.zip › animals-3869357-supplementary.pdf]

Supplementary materials

Table S1. Modified staining protocols, AB-PAS and MC, used in our study to identify mucosubstances and goblet cells (GCs).

Solution	Duration
**Xylene I**	00:03:00
**Xylene II**	00:03:00
**Xylene III**	00:03:00
**Ethanol 100%**	00:02:00
**Ethanol 96%**	00:02:00
**Ethanol 70%**	00:02:00
**Ethanol 60%**	00:02:00
**Aqua dest.**	00:02:00
**3% acetic acid**	00:03:00
**Alcian blue 1% (pH 2,5)**	00:60:00
**3% acetic acid**	00:01:00
**running tap water**	00:03:00
**Aqua dest.**	00:01:00
**Periodic acid 1%**	00:10:00
**running tap water**	00:03:00
**Aqua dest.**	00:01:00
**Schiff's reagent**	00:10:00
**running tap water**	00:03:00
**Aqua dest.**	00:01:00
**Hematoxylin (after Mayer)**	00:00:15
**running tap water**	00:05:00
**Ethanol 70%**	00:02:00
**Ethanol 96%**	00:02:00
**Ethanol 100%**	00:02:00
**Xylene I**	00:05:00
**Xylene II**	00:05:00
**Xylene III**	00:05:00
**Mounting medium Entellan™**	00:01:00
**Total duration of staining**	02:22:15

Solution	Duration
**Xylene I**	00:03:00
**Xylene II**	00:03:00
**Xylene III**	00:03:00
**Ethanol 100%**	00:02:00
**Ethanol 96%**	00:02:00
**Ethanol 70%**	00:02:00
**Aqua dest.**	00:02:00
**Hematoxylin**	00:01:00
**running tap water**	00:10:00
**Aqua dest.**	00:01:00
**Mucicarmine after Mayer (1:5)**	00:60:00
**running tap water**	00:02:00
**Aqua dest.**	00:01:00
**Ethanol 70%**	00:01:00
**Metanil Yellow 2%**	00:03:00
**running tap water**	00:02:00
**Ethanol 70%**	short
**Ethanol 96%**	short
**Ethanol 100%**	short
**Xylene I**	00:05:00
**Xylene II**	00:05:00
**Xylene III**	00:05:00
**Mounting medium Entellan™**	00:01:00
**Total duration of staining**	01:53:00

Alcian Blue–Periodic Acid–Schiff (AB–PAS) is the most widely used pan-mucin stain. Here, we extend this approach by systematically comparing mucicarmine (MC) in porcine gastrointestinal tissue. Both methods delineate goblet cells (GCs): AB (pH 2.5) and MC detect acidic mucins, whereas neutral mucins are AB-negative but PAS- and MC-positive. On AB–PAS, acidic mucins stain blue, neutral magenta, and mixed mucins purple. To our knowledge, this is the first application of MC to quantify goblet cell density in porcine intestine.

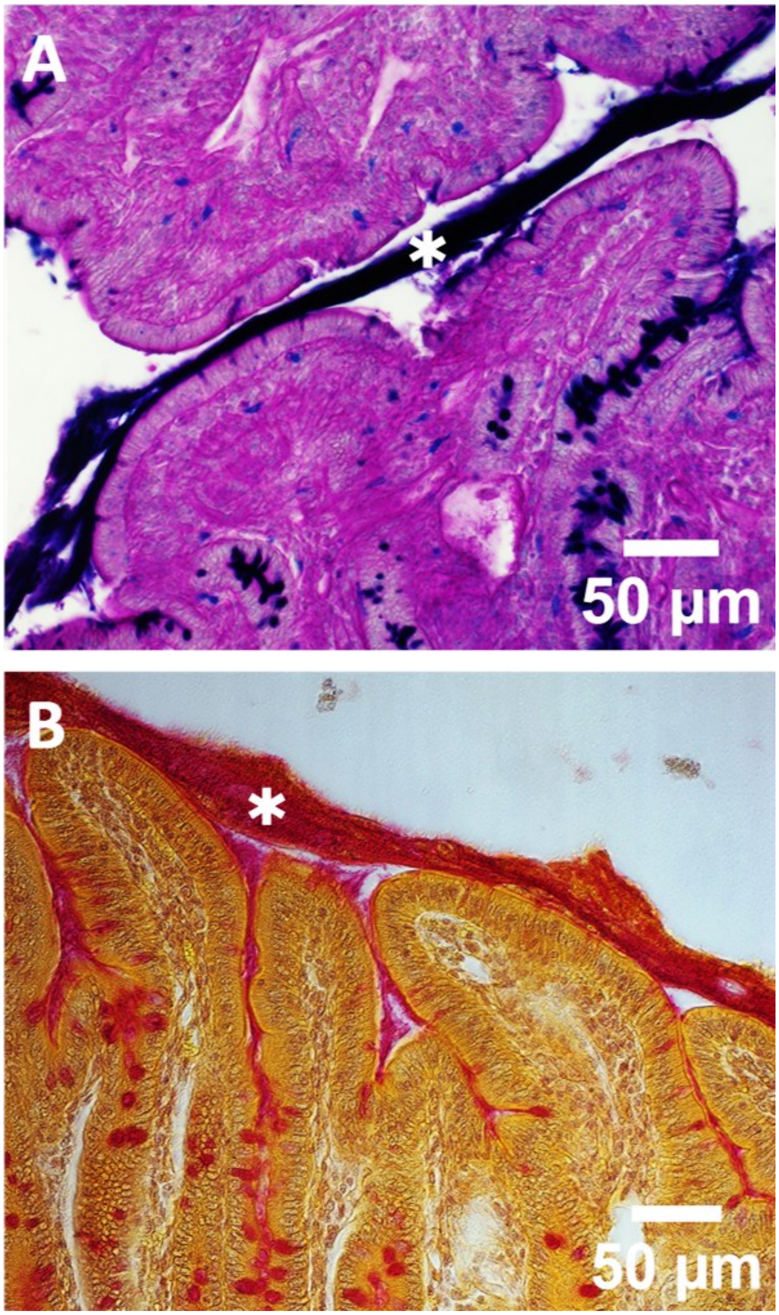

Figure S1. Porcine jejunum—luminal mucus gel layer. (**A**) Alcian Blue–Periodic Acid–Schiff (AB–PAS); (**B**) Mucicarmine. Both stains highlight the luminal mucus gel layer (asterisks) overlying intact villous epithelium, showing the characteristic small-intestinal pattern: the layer is comparatively thin, irregular, and concentrated at villus tips, and may be discontinuous between adjacent villi. In AB–PAS (**A**), goblet-cell mucins within the epithelium appear purple and the surface gel forms a dark-purple luminal coating; in mucicarmine (**B**), the gel appears as a dense deep-red band draped over villus tips with uneven thickness. Physiologically, the jejunal mucus forms a single, loose, MUC2-based gel that partially fills intervillous spaces and usually caps villus tips; and thinner/more heterogeneous than colonic mucus. (*Scale Bar* = 50 µm).

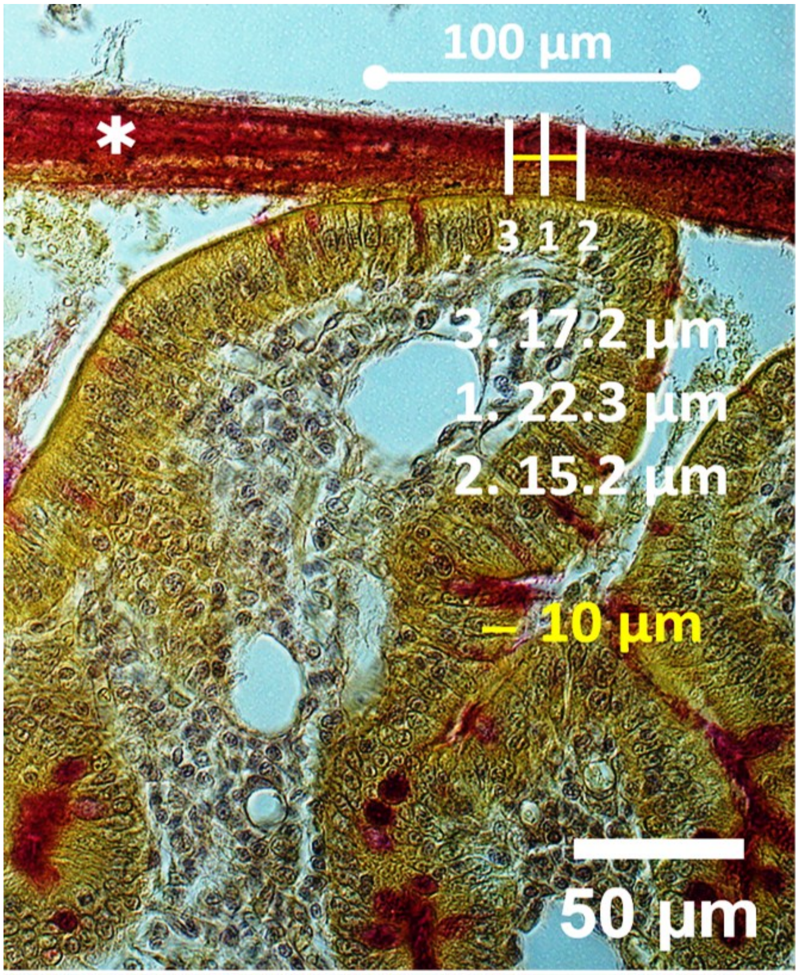

Results

File Edit Font Results

	Area	Mean	StdDev	Min	Max	Angle	Length
1	16.949	9935.309	377.820	8728.879	11121.546	-89.421	100.260
2	3.789	10337.363	297.088	9534.546	11125.000	-87.397	22.302
3	1.738	10470.033	332.560	9821.000	11255.000	-90.000	10.127
4	2.592	10360.489	343.505	9616.000	11195.800	-86.186	15.224
5	1.738	10098.033	438.219	9126.000	11081.000	-90.000	10.127
6	2.934	10230.264	323.708	9351.706	11103.883	-86.634	17.245

Figure S2. Ileum (mucicarmine)—measurement of the mucus gel layer. Light micrograph of porcine small-intestinal mucosa (ileal villus) stained with mucicarmine. The luminal mucus gel layer appears as a deep-red band and is marked with an asterisk (left), overlying intact villous epithelium. Thickness was measured in ImageJ using three adjacent, perpendicular line profiles (white) drawn from the epithelial surface into the mucus and spaced 10 µm apart (yellow label). The corresponding measurements are shown next to each profile: 22.3 µm (1), 15.2 µm (2), and 17.2 µm (3). The field meets the pre-specified inclusion criterion of a continuous mucus layer ≥ 100 µm in length along intact epithelium, illustrated by the 100 µm reference ruler at the top. The ImageJ "Results" table at the bottom documents the measurement outputs and metadata *(Scale Bar = 50 µm)*.

Table S2. Cytokeratin 18 (CK18) indirect immunofluorescence staining protocol.

Instruction	Material	Duration
Fix on a sterile slide	1:1 Acetone / Methanol (at -20°C)	5 min. at RT
Rinse	0,1 % Tween-20 in TBS	5 x 7 min.
Slides in humidity staining chamber		
Permeabilize	0,1 % Triton in 0,1 % Tween-20 in TBS	5 min. at RT
Rinse and rimming of every section with Pap-Pen	0,1 % Tween-20 in TBS	5 x 7 min.
Block with Blocking Buffer	NGS 1:100 in 0,1 % Tween-20 in TBS + 1 % BSA	1 hour at RT
Rinse	0,1 % Tween-20 in TBS	5 x 7 min.
Primary antibodies:<br>Mouse Anti-Cytokeratin Peptide CK18 monoclonal – unconjugated IgG1 (C8541) CY - 90	in 0,1 % Tween-20 in TBS + 1 % BSA 1:10.000	overnight at 4°C in dark
Rinse	0,1 % Tween-20 in TBS	5 x 7 min.
Secondary Antibodies:<br>Goat anti-mouse IgG1 A488 Invitrogen A21121 polyclonal IgG, Ex 499 nm, Em 520 nm	in 0,1 % Tween-20 in TBS + 1 % BSA 1:200	1 hour in dark at RT
Rinse	0,1 % Tween-20 in TBS	5 x 7 min.
Nuclear staining	1:10 DAPI in 0,1 % Tween-20 in TBS	5 min.
Rinse	0,1 % Tween-20 in TBS	5 x 7 min.
Mounting	Mowiol®	/

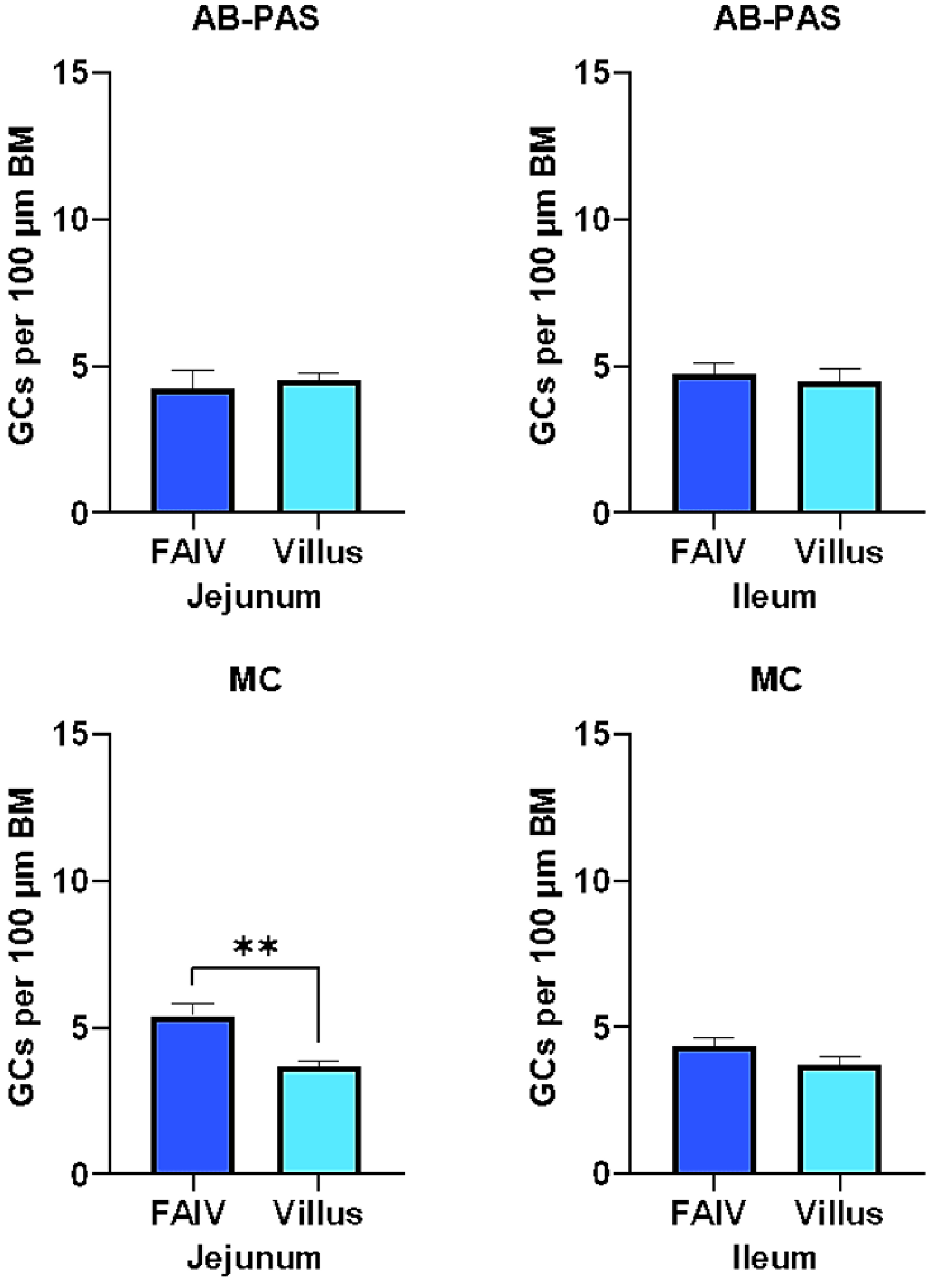

Figure S3. Comparison of the total relative number of goblet cells (GCs) per 100 µm of basement membrane (BM) between the FAIV and regular villi in either the jejunum or ileum. All the data were normally distributed and analyzed using Student's *t*-test. ($n = 6$; ns $p \geq 0.05$; *$p < 0.05$; **$p < 0.01$; ***$p < 0.001$). Abbreviations: FAIV, follicle-associated intestinal villus; AB-PAS, Alcian Blue–Periodic Acid–Schiff; MC, mucicarmine.
